# microRNA Crosstalk Influences Epithelial-to-Mesenchymal, Endothelial-to-Mesenchymal, and Macrophage-to-Mesenchymal Transitions in the Kidney

**DOI:** 10.3389/fphar.2019.00904

**Published:** 2019-08-16

**Authors:** Swayam Prakash Srivastava, Ahmad F. Hedayat, Keizo Kanasaki, Julie E. Goodwin

**Affiliations:** ^1^Department of Pediatrics, Yale University School of Medicine, New Haven, CT, United States; ^2^Internal Medicine 1, Shimane University Faculty of Medicine, Izumo, Japan

**Keywords:** microRNAs, diabetic kidney disease, kidney fibrosis, microRNA crosstalk, epithelial-to-mesenchymal transition, endothelial-to-mesenchymal transition, macrophage-to-mesenchymal transition

## Abstract

microRNAs (miRNAs) are small, non-coding nucleotides that regulate diverse biological processes. Altered microRNA biosynthesis or regulation contributes to pathological processes including kidney fibrosis. Kidney fibrosis is characterized by deposition of excess extracellular matrix (ECM), which is caused by infiltration of immune cells, inflammatory cells, altered chemokines, and cytokines as well as activation and accumulation of fibroblasts in the kidney. These activated fibroblasts can arise from epithelial cells *via* epithelial-to-mesenchymal transition (EMT), from bone marrow-derived M2 phenotype macrophages *via* macrophage-to-mesenchymal transition (MMT), from endothelial cells *via* endothelial-to-mesenchymal transition (EndMT), from resident fibroblasts, and from bone marrow-derived monocytes and play a crucial role in fibrotic events. Disrupted microRNA biosynthesis and aberrant regulation contribute to the activation of mesenchymal programs in the kidney. miR-29 regulates the interaction between dipeptidyl peptidase-4 (DPP-4) and integrin β1 and the associated active transforming growth factor β (TGFβ) and pro-EndMT signaling; however, miR-let-7 targets transforming growth factor β receptors (TGFβRs) to inhibit TGFβ signaling. N-acetyl-seryl-aspartyl-lysyl-proline (AcSDKP) is an endogenous anti-fibrotic peptide, which is associated with fibroblast growth factor receptor 1 (FGFR1) phosphorylation and subsequently responsible for the production of miR-let-7. miR-29 and miR-let-7 family clusters participate in crosstalk mechanisms, which are crucial for endothelial cell homeostasis. The physiological level of AcSDKP is vital for the activation of anti-fibrotic mechanisms including restoration of anti-fibrotic microRNA crosstalk and suppression of profibrotic signaling by mitigating DPP-4-associated mesenchymal activation in the epithelial cells, endothelial cells, and M2 phenotype macrophages. The present review highlights recent advancements in the understanding of both the role of microRNAs in the development of kidney disease and their potential as novel therapeutic targets for fibrotic disease states.

## Origin of Fibroblasts in Kidney

**Figure 1:**
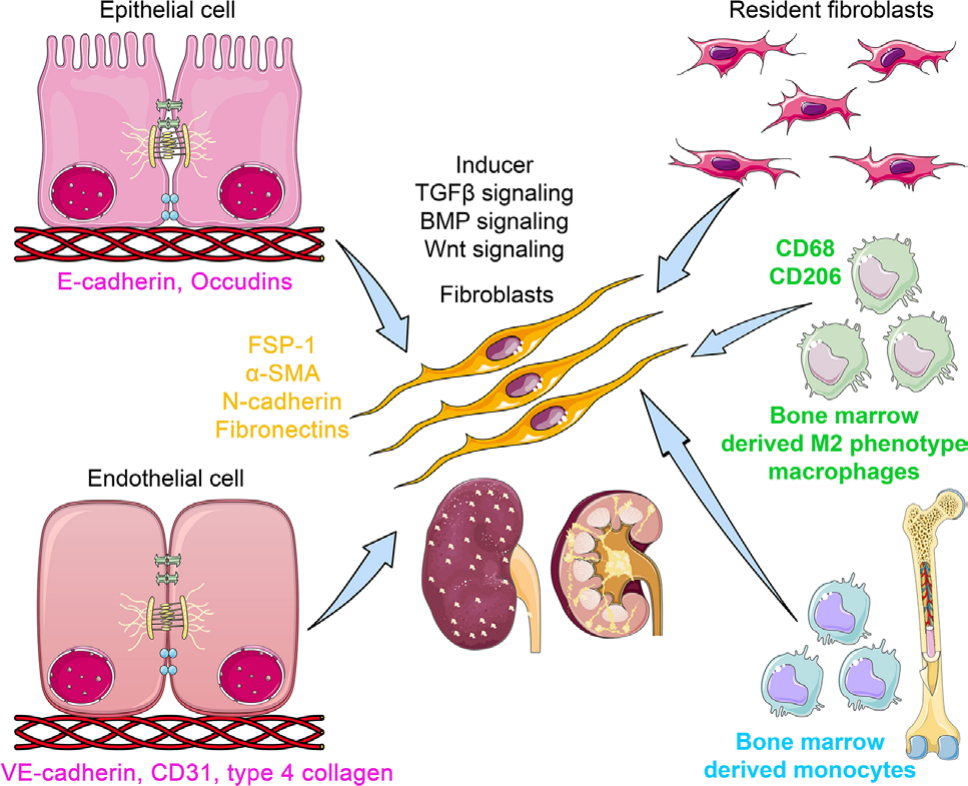
The origins of kidney myofibroblasts. Myofibroblasts are originated from resident fibroblasts, bone marrow-derived monocytes, bone marrow-derived M2 phenotype macrophages *via* macrophage-to-mesenchymal transition (MMT) process, from epithelial cell *via* epithelial-to-mesenchymal transition (EMT) program, and from endothelial cells *via* endothelial to mesenchymal transition (EndMT) program. During the process of EMT, epithelial cells loose the epithelial markers (E-cadherin and occludins) and gain the mesenchymal markers (FSP-1, α-SMA, N-cadherin, and fibronectin). In the EndMT process, endothelial cells lose the endothelial markers (CD31 and VE cadherin) and gain the mesenchymal markers. However, during the process of MMT, the myo-fibroblasts co-express M2 phenotype macrophage markers (CD206) with mesenchymal markers. TGFβ, BMP, and Wnt signaling play a crucial role in the activation of mesenchymal transition processes. The myofibroblasts are polar, pointed, and elongated in shape and ability to migrate and invade the neighbor cells.

Kidney fibrosis is the final outcome of progressive diabetic kidney disease that can lead to end stage renal disease (ESRD) (Parving, 2001; Remuzzi et al., 2002; Alicic et al., 2017; Isaka, 2018; Luyckx et al., 2018; Umanath and Lewis, 2018; Allison, 2019; Cooper and Warren, 2019; Djudjaj and Boor, 2019). It results in the massive destruction of cellular structures and kidney function. Kidney fibrosis is caused by prolonged injury and deregulation of normal wound healing processes in association with excess deposition of extracellular matrix (ECM) (Lee and Kalluri, 2010; Nogueira et al., 2017). In such fibrotic processes, kidney fibroblasts play vital roles, but the origin of fibroblasts still remains unclear and a matter of ongoing debate (Kanasaki et al., 2013a; El Agha et al., 2017; Di Carlo and Peduto, 2018). These debates were based on each report that stick to the idea that one single cell type can explain majority of fibrogenesis events in kidney; renal fibrogenesis is the consequence of the interaction between all the cell types in kidney, either kidney cells or invaded inflammatory cells (Liu, 2011; Medici and Kalluri, 2012; Lebleu et al., 2013; Mack and Yanagita, 2015; Nogueira et al., 2017). Also, complete conversion into mesenchymal cell types is not essential; intermediate phenotypes of mesenchymal programs were sufficient to induce alteration in fibrogenic programs (Kanasaki et al., 2013a; Lebleu et al., 2013; Kim et al., 2017; Xing and Tian, 2019). Activation of resident fibroblasts is the first step in renal fibrogenesis (Grgic et al., 2012; Sato and Yanagita, 2017). **Figure 1** depicts the sources of fibroblasts that have been proposed, including from pericytes, fibrocytes, bone marrow-derived monocytes, and fibroblast originating from bone marrow-derived M2 type macrophages *via* macrophage-to-mesenchymal transition (MMT), fibroblasts originating from epithelial-to-mesenchymal transition (EMT), and endothelial-to-mesenchymal transition (EndMT) (Barnes and Gorin, 2011; Lebleu et al., 2013; Yan et al., 2016; Sato and Yanagita, 2017; Di Carlo and Peduto, 2018; Xiong et al., 2018; Glover et al., 2019). The available treatments for kidney fibrosis are unsatisfactory to address this problem, and approved therapies are not cell-specific in nature (Lee et al., 2015; Quiroga et al., 2015; Breyer and Susztak, 2016). Current treatment strategies may slow the rate of disease progression but cannot prevent progression to ESRD (Brenner et al., 2001; Lee et al., 2015; Quiroga et al., 2015; Johnson et al., 2016; Luyckx et al., 2018); hence, current therapies are ineffective.

## EMT in Renal Fibrosis

EMT involves a series of events through which epithelial cells lose their epithelial characteristics and acquire properties of typical mesenchymal cells (Hills and Squires, 2011; Grande et al., 2015; Lovisa et al., 2015; Marquez-Exposito et al., 2018). **Figure 1** displays the unique phenotypes of epithelial, endothelial, and mesenchymal cells. Epithelial cells are normally associated tightly with their neighbor cells, which inhibits their potential to dissociate from the epithelial layer. In contrast, mesenchymal cells do not form a layer of cells or intercellular adhesion complexes (Srivastava et al., 2013). Mesenchymal cells are elongated in shape and exhibit end-to-end polarity and focal adhesions, allowing for increased migratory capacity (Srivastava et al., 2013). In adults, the main function of fibroblasts, which are prototypical mesenchymal cells that exist in many tissues, is to maintain structural integrity by secreting extracellular matrix (ECM). Fibroblast-specific protein 1 (FSP-1; also known as S100A4), alpha-smooth muscle actin (αSMA), fibronectin, and collagen I have proved to be reliable markers to characterize the mesenchymal products generated by EMT that occurs during the development of fibrosis in various organs (Kalluri and Weinberg, 2009; Srivastava et al., 2013; Alidadiani et al., 2018). Inflammatory injury to the mouse kidney can result in the recruitment of a diverse array of cells that can trigger EMT through their release of growth factors, such as transforming growth factor-beta (TGFβ), platelet-derived growth factor (PDGF), epidermal growth factor (EGF), and fibroblast growth factor-2 (FGF-2) (Kalluri and Weinberg, 2009; Alidadiani et al., 2018; Liu et al., 2019b).

## EndMT in Renal Fibrosis

Vascular endothelial cells can also originate fibroblasts by undergoing a phenotypic transition, referred to as EndMT (Srivastava et al., 2013; Curci et al., 2014; Li et al., 2017; Lovisa and Kalluri, 2018; Glover et al., 2019). **Figure 1** displays the process and contribution of EndMT to fibrogenesis. EndMT is the process that is characterized by the loss of endothelial markers, including cluster of differentiation 31 (CD31) and vascular endothelial cadherin (VE-cadherin), and acquisition of the expression of mesenchymal proteins including αSMA (Zeisberg et al., 2007; Srivastava et al., 2013; Curci et al., 2014; Lovisa and Kalluri, 2018; Glover et al., 2019). EndMT contributes to cardiac fibrogenesis (Zeisberg et al., 2007; Kovacic et al., 2019), pulmonary fibrosis (Good et al., 2015; Cho et al., 2018), idiopathic hypertension (Kitao et al., 2009; Ranchoux et al., 2015), and fibrosis in the cornea (Nakano et al., 2008; Medici, 2016; Lee et al., 2018). Many signaling pathways that govern EMT also regulate EndMT in the embryonic heart, during the development of cardiac fibrosis (Pardali et al., 2017; Man et al., 2019) and pulmonary fibrosis (Guan and Zhou, 2017; Pardali et al., 2017), and in liver fibrogenesis (Dufton et al., 2017; Pardali et al., 2017). Compared to EMT, comparatively little is known about EndMT. The contribution of EndMT to renal fibrosis has been reviewed in recent years (Srivastava et al., 2013; Curci et al., 2014; Medici, 2016; Glover et al., 2019). In the adult organism, pathological conditions such as injury, inflammation, or aging can induce EndMT and influence organ fibrosis (Cho et al., 2018). Zeisberg et al. (2008) performed a seminal experiment that confirmed the contribution of EndMT in renal fibrosis in three mouse models: unilateral ureteral obstruction (UUO), a surgical model used to study progressive tubulointerstitial fibrosis, streptozotocin (STZ)-induced diabetic mice, and α3 chain of collagen type 4 (COL4A3) knockout mice (a mouse model for Alport syndrome). The authors reported that a considerable number of myofibroblasts co-expressed CD31 with αSMA and FSP-1 in all three models (Zeisberg et al., 2008). The authors analyzed the kidneys of diabetic CD-1 mice 6 months after a single injection of STZ and showed that kidneys had progressive glomerular sclerosis and tubulointerstitial fibrosis. The co-immunofluorescence analysis in the kidneys of diabetic CD-1 mice displayed approximately 40% of all FSP-1 positive cells, and 50% of αSMA positive stromal cells were CD31-positive (Zeisberg et al., 2008). Similarly, in the kidneys of COL4A3 knockout mice, 45% of all αSMA-positive fibroblasts and 60% of all FSP-1-positive fibroblasts were CD31-positive, suggesting that these fibroblasts are of endothelial origin and that EndMT might contribute critically to the development and progression of renal fibrosis (Zeisberg et al., 2008). Li et al. (2009) confirmed that EndMT contributes to the activation of myofibroblasts in early diabetic renal fibrosis. In the landmark experiment using endothelial cell-lineage tracing with *Tie2*-Cre and LoxP-enhanced green fluorescent protein (EGFP) transgenic mice, the authors confirmed a large population of interstitial αSMA-positive cells of endothelial origin in the fibrotic kidneys of STZ-induced diabetic mice (Li et al., 2009). These endothelial cells demonstrated a set of biomarkers including VE-cadherin, CD31, tyrosine kinase with immunoglobulin-like EGF-like domains 1 (TIE1), TEK receptor kinase (TIE2), von Willebrand factor (vWF), and cytokeratins (Srivastava et al., 2013). During the process of EndMT, biochemical changes leads to the decreased expression of endothelial markers and the gain of mesenchymal markers such as FSP-1, αSMA, smooth muscle 22-alpha (SM22α), N-cadherin, fibronectin, vimentin, type I and III collagen, nestin, cluster of differentiation 73 (CD73), matrix metalloproteinase -2 (MMP-2), and matrix metalloproteinase-9 (MMP-9) (Medici and Kalluri, 2012; Srivastava et al., 2013; Srivastava et al., 2016).

## MMT in Renal Fibrosis

Interstitial fibrosis is the key characteristics in chronic renal allograft injury (Boor and Floege, 2015; Bontha et al., 2017). In the chronic renal allograft injury, diverse ranges of immune and nonimmune responses cause the macrophages to undergo macrophage-to-mesenchymal transition (MMT) process (Wang et al., 2019; Zhou et al., 2019). Higher rate of MMT contributes in the development of interstitial fibrosis (Wang et al., 2017). Wang et al. performed the seminal experiments on MMT and identified that the kidneys in the patients and in the experimental chronic renal allograft injury displayed co-expression of macrophage marker (CD68) with myofibroblast marker (α-SMA) (Wang et al., 2017). Approximately 50% cells of total myo-fibroblasts cells in the kidneys were CD68^+^/α-SMA^+^ and were associated with interstitial fibrosis after the chronic renal allograft injury (Wang et al., 2017). Moreover, MMT processes were observed mainly in the bone marrow-derived M2-phenotype macrophages (Wang et al., 2017). However, M1-phenotype macrophages are responsible for pro-inflammatory cytokine production and contribute in the graft loss in the kidneys (Ma et al., 2013; Kwan et al., 2014; Salehi and Reed, 2015). These data are in accord with previous observation that showed that bone marrow-derived monocytes and macrophages can contribute in the collagen formation by inducing the MMT processes in the kidneys of mouse model of ureteric obstruction and in the progressive chronic kidney disease (CKD) subjects (Yang et al., 2013; Liu et al., 2018). The MMT processes were dependent on TGFβ-smad3 signaling (Wang et al., 2017). However, M2-to-M1 phenotype conversion can induce cytokines that lead to the higher MMT process and can be TGFβ independent (Wang et al., 2017).

## MicroRNAs Regulate EMT and EndMT

MicroRNAs (miRNAs) are well known for their regulatory role in diseases like diabetes, cancer, and fibrosis (Ruiz and Chakrabarti, 2013; Srivastava et al., 2013; Shah et al., 2016; Miao et al., 2018; Nadeem et al., 2018; Tan et al., 2018). They are small (around 22 nt) evolutionarily conserved, non-coding RNAs that regulate the expression of protein coding genes at the post-transcriptional level by binding to regions complementary to the 3’untranslated regions (UTR) of target mRNA. miRNAs suppress protein expression by either inhibiting mRNA translation or facilitating mRNA degradation (Kaur et al., 2011; Gebert and Macrae, 2019). Differential expression in tissues and tissue-specific selectivity enable them to play an important role in understanding the pathophysiology as well as the potential therapy of kidney diseases (Lv et al., 2018; Nascimento and Domingueti, 2019; Zhao et al., 2019). Modulation of kidney-specific miRNAs may enable renal-specific expression of target proteins that are vital for kidney function (Metzinger-Le Meuth et al., 2019).

**Figure 2:**
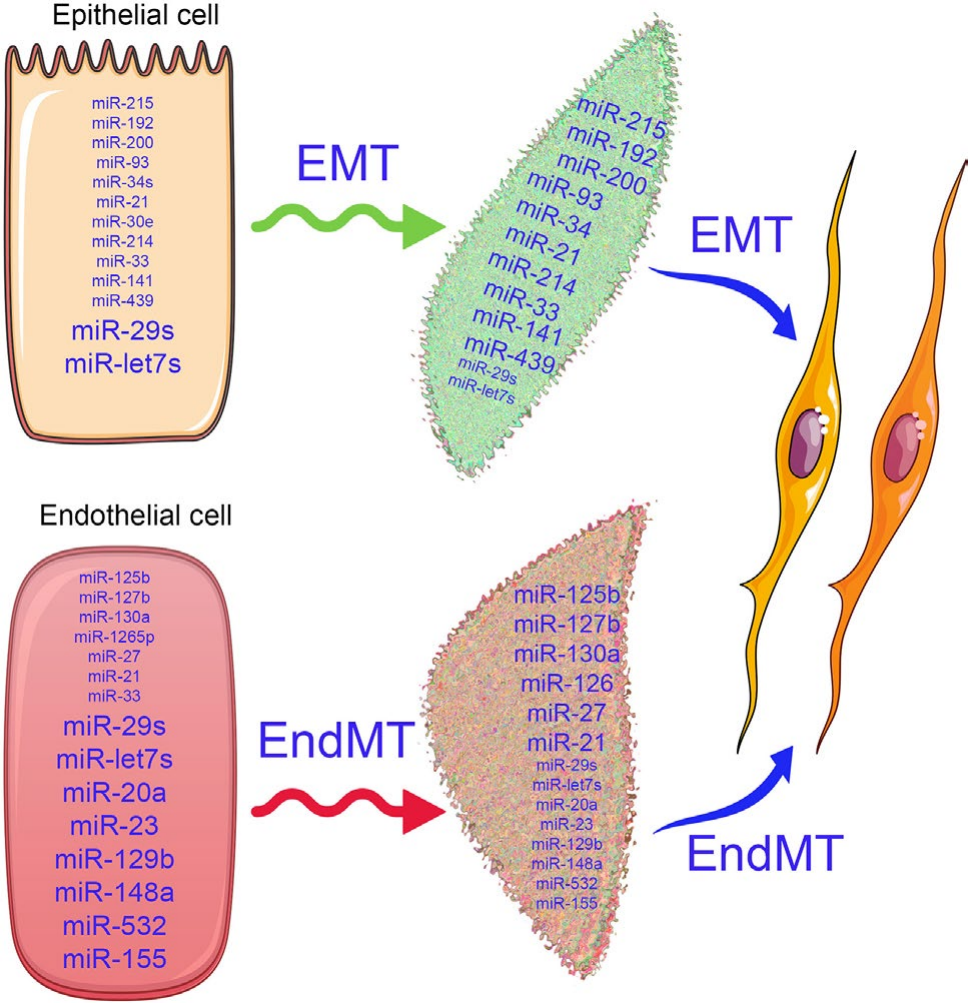
Alterations in microRNA expression influence the EMT and EndMT programs. In the epithelial cells, the antifibrotic miRNAs (miR-29 and let-7s) and profibrotic microRNAs (miR-15, miR-192, miR-200, miR-293, miR-34, miR-33, miR-214, miR-439, and miR-141) are expressed physiologically. Similarly, in the endothelial cells, antifibrotic microRNAs (miR-29, let-7, miR-20, miR-23, miR-129b, miR-148a, miR-532, and miR-155) and profibrotoic microRNAs (miR-125b, miR-127b, miR-130a, miR-27, miR-33, miR-21, and miR-1265) are expressed. The differential expressions of microRNAs in the epithelial cells and endothelial cells regulate the biological pathways and signaling events and maintain the homeostasis. As depicted by changes in font size, when healthy cells undergoes the mesenchymal transition process, the expression of anti-fibrotic microRNAs decreases, while pro-fibrotic microRNA expression increases and disrupts the cellular homeostasis.

Differential miRNA expression data suggest a role of altered miRNA in the pathogenesis of kidney disease (Bhatt et al., 2011; Lorenzen et al., 2011; Zhong et al., 2011; Chau et al., 2012; Chung and Lan, 2015; Van Der Hauwaert et al., 2015; Schauerte et al., 2017; Zhang et al., 2017; Hajarnis et al., 2018; Thomas et al., 2018; Xi et al., 2018; Yang et al., 2018; Zheng et al., 2018; Fujii et al., 2019; Liu et al., 2019a; Liu et al., 2019c; Zhao et al., 2019). The term fibromiR has been suggested for those miRNAs that regulate fibro-proliferative diseases (Pottier et al., 2014). So far, researches in this area have included TGFβ-associated regulation of miRNA expression in diabetic nephropathy (Kato et al., 2007; Kato et al., 2010; Kato et al., 2011; Kolling et al., 2017; Zanchi et al., 2017; Assmann et al., 2018; Zhang et al., 2018; Nascimento and Domingueti, 2019; Regmi et al., 2019), p53 induction of miR-34a in ischemic acute kidney injury (Bhatt et al., 2010), and miR-15a regulation of the cell division cycle regulator Cdc25A (Lee et al., 2008). Natarajan and colleagues reported that TGFβ-induced up-regulation of miR-192, miR-216a, and miR-217 in a diabetic mouse model and in glomerular mesangial cells (Kato et al., 2007; Kato et al., 2010) *via* targeting smad interacting protein 1 (SIP1), protein-tyrosine phosphatase (PTEN), and y-box binding protein 1 (Ybx1) played critical roles in collagen expression (Kato et al., 2007; Kato et al., 2010). However, clinical studies of diabetic nephropathy display remarkably lower miR-192 expression; further studies are required to explain this discrepancy (Krupa et al., 2010). In other studies, miR-335 and miR-43a encourage renal cell senescence by suppressing mitochondrial antioxidative enzymes (Bai et al., 2011). miR-192 has been shown to mediate lysine deficient protein kinase 1 (WNK1)-regulated sodium and potassium balance (Elvira-Matelot et al., 2010) and TGFβ-induced fibrosis (Chung et al., 2010). Moreover, angiotensin-converting-enzyme inhibitor (lisinopril) treatment caused an anti-fibrotic effect in the kidneys of Munich Wistar Fromter rats (a mouse model of progressive nephropathy) by inhibiting miR-324-3p-dependent suppression of prolyl endopeptidase (POP), a serine peptidase involved in the synthesis of the endogenous antifibrotic peptide AcSDKP, which is critical in the homeostasis of ECM secretion (Macconi et al., 2012). A recent study demonstrated that a feedback loop between miR-21 and programmed cell death protein 4 (PDCD4) and activated protein (AP-1) drives progression in a mouse model of renal fibrosis (Sun et al., 2018). A significant number of reviews have addressed the role of miRNAs in renal fibrosis (Li et al., 2010; Amrouche et al., 2011; Lorenzen et al., 2011; Chandrasekaran et al., 2012; Srivastava et al., 2013; Chung and Lan, 2015; Kato and Natarajan, 2015; Van Der Hauwaert et al., 2015; Zhang et al., 2017; Assmann et al., 2018; Lv et al., 2018; Fujii et al., 2019; Nascimento and Domingueti, 2019; Regmi et al., 2019; Zhao et al., 2019). miRNA actions can be pro-fibrotic or anti-fibrotic depending on the kidney cell type. **Figure 2** depicts the altered level of miRNAs in EMT and EndMT processes, which regulates fibroblast synthesis and fibroblast accumulation in kidney.

## Anti-Fibrotic microRNAs in the Kidney

### miR-29 Family

**Figure 3:**
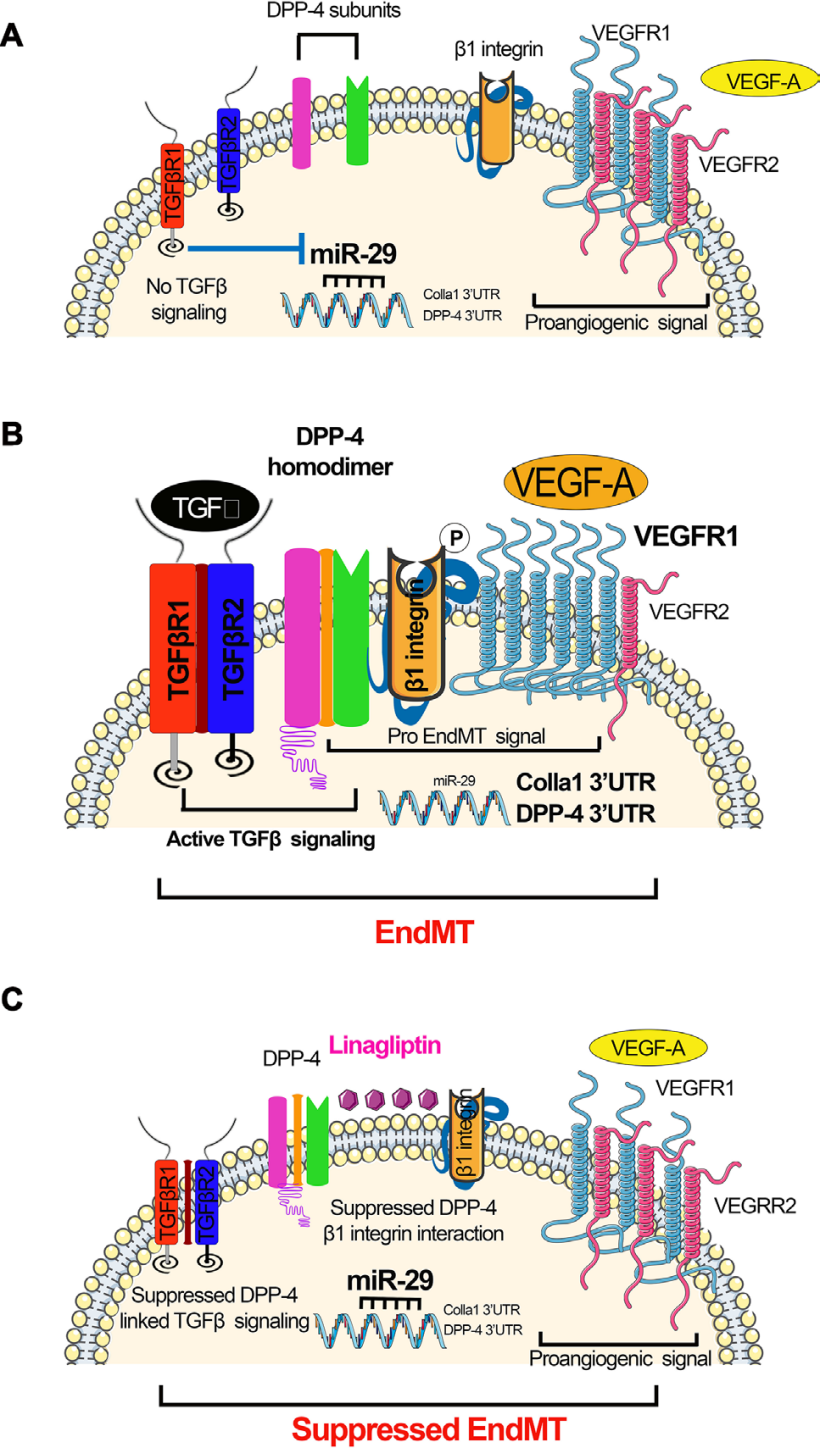
miR-29s regulate DPP-4–integrin β1-associated active TGFβ signaling and switch between VEGFR1 and VEGFR2 in the endothelial cells. **(A)** Absent TGFβ signaling; in the absence of TGFβ, miR-29 families were expressed at normal level, which targets the 3’UTR of DPP-4 mRNA and 3’UTR of Colla 1 mRNA. Suppressed level of DPP-4–β-integrin further leads to suppression in the TGFβ signaling. **(B)** Active TGFβ signaling resulting in EndMT. Active TGFβ signaling causes suppression in the miR-29, which results to the higher DPP-4 mRNA and Colla1 transcription. Higher level of DPP-4–β-integrin and VEGF influences the active TGFβ signaling. **(C)** Suppressed TGFβ signaling mediated by DPP-4 inhibitor (linagliptin) restores miR-29 expression level and reduces the level of VEGF. The elevated level of miR-29 causes the suppression of the DPP-4–β-integrin level. The concomitant effect of reduced level of VEGF suppressed level DPP-4–β-integrin and finally leads to inhibition of active TGFβ signaling and suppression in the EndMT processes.

The miR-29 family clusters emerge as a major anti-fibrotic player in kidney fibrosis associated with *Smad*-dependent and *Smad*-independent pathways (Chung and Lan, 2015). The expression level of members of miR-29 family is significantly suppressed in both renal fibrosis (Lan, 2012; Meng et al., 2013; Srivastava et al., 2014) and diabetic (Srivastava et al., 2016) and hypertensive nephropathy (Wei et al., 2013). miR-29 is downstream of *Smad3* and can suppress the upstream TGFβ–Smad3 signaling by miR-29b-mediated negative feedback (He et al., 2013). miR-29b binds to the coding region of TGFβ1 mRNA at exon 3, which blocks the translation of TGFβ1, resulting in the suppression of *Smad3*-dependent fibrosis (Zhang et al., 2014). miR-29 binds to the promoter region of smad3 and exerts anti-fibrotic properties. In vitro, overexpression of miR-29 inhibited, but knockdown of miR-29 enhanced, TGFβ1-induced expression of collagens I and III in cultured proximal tubular epithelial cells (TECs) (Qin et al., 2011; Wang et al., 2012a; Qi and Yang, 2018). However, ultrasound-mediated gene delivery of miR-29 blocked progressive renal fibrosis in obstructive nephropathy (UUO) (Qin et al., 2011; Qi and Yang, 2018). Data from various studies have shown that members of the miR-29 family target different isoforms of collagen and have an anti-fibrotic role (Wang et al., 2012a; Qi and Yang, 2018). TGFβ1 inhibits the beneficial role of miR-29 family by down-regulating the expression in TECs (Du et al., 2010; Wang et al., 2012a), mesangial cells (Wang et al., 2012a), and podocytes (Wang et al., 2012a). miR-29b suppression contributes to progressive renal injury in several mouse models of chronic kidney disease (CKD) (Qin et al., 2011; Wang et al., 2012a; Ramdas et al., 2013); however, overexpression of miR-29b provides a therapeutic benefit in UUO and db/db mice (Qin et al., 2011; Chen et al., 2014a). In db/db mice, miR-29a has been shown to be elevated in the liver and regulate gluconeogenesis (Pandey et al., 2011). Of note, treatment of rats with losartan caused a remarkable increase in the level of miR-29b expression, which was linked with lower expression of collagen, fibronectin, and laminin, and provided protection from kidney fibrosis (Wang et al., 2012a). miR-29 family clusters also inhibit elevated dipeptidyl dipeptidase-4 (DPP-4) protein levels by targeting the 3’UTR of its mRNA (Kanasaki et al., 2014; Shi et al., 2015). TGFβ2-mediated induction of DPP-4 and down-regulation of miR-29 are associated with EndMT (Kanasaki et al., 2014; Shi et al., 2015). miR-29 and TGFβ signaling exhibit a negative feedback loop and regulate each other, as induction of TGFβ signaling suppresses downstream miR-29 (Kanasaki et al., 2014) and miR-29 suppresses upstream TGFβ signaling (Zhang et al., 2014), This relationship is quite interesting and supports an anti-fibrotic role of miR-29 in kidney fibrosis. The schematic diagram displays the renal protective action of miR-29 in EndMT and associated renal fibrosis (**Figure 3**).

A new pro-fibrotic molecular mechanism exists, which is associated with the interaction between DPP-4 and integrin β1 and is a therapeutic target for kidney fibrosis during diabetes (Shi et al., 2015). In endothelial cells, miR-29 negatively regulates the DPP-4 and integrin β1 interaction (Shi et al., 2015). This interaction is a key regulator of the switch between vascular endothelial growth factor 1 (VEGFR1) and vascular endothelial growth factor 2 (VEGFR2) (Shi et al., 2015). VEGFR1 is a positive effector of monocyte and macrophage migration and has been reported as a negative regulator of the VEGFR2 signaling capacity of VEGF-A (Olsson et al., 2006). Integrin β1 is involved in several biological processes, including cell migration, cell adhesion, formation of basement membrane, and control of cell cycle (Mulrooney et al., 2001; Tanjore et al., 2008; Kanasaki et al., 2013b). Decreased expression of DPP-4 or integrin β1 inhibits TGFβ2-stimulated heterodimer formation of transforming growth factor β receptors (TGFβRs), thereby abolishing active TGFβ signaling (**Figure 3A**). Increased expression of TGFβ causes suppression of miR-29 (Qin et al., 2011) and increases the interaction between DPP-4 and integrin β1-induced VEGFR1 expression level with concomitant reduction of VEGFR2 expression levels, leading to active TGFβ and pro-EndMT signaling (**Figure 3B**). The DPP-4 inhibitor linagliptin is associated with EndMT inhibition by suppressing the interaction between DPP-4 and integrin β1 and elevating the miR-29 level (Kanasaki et al., 2014). TGFβ2 increases VEGFR1 levels, and TGFβ2-induced up-regulation of VEGFR1 can be suppressed by linagliptin (**Figure 3C**).

### miR-let-7 Family

**Figure 4:**
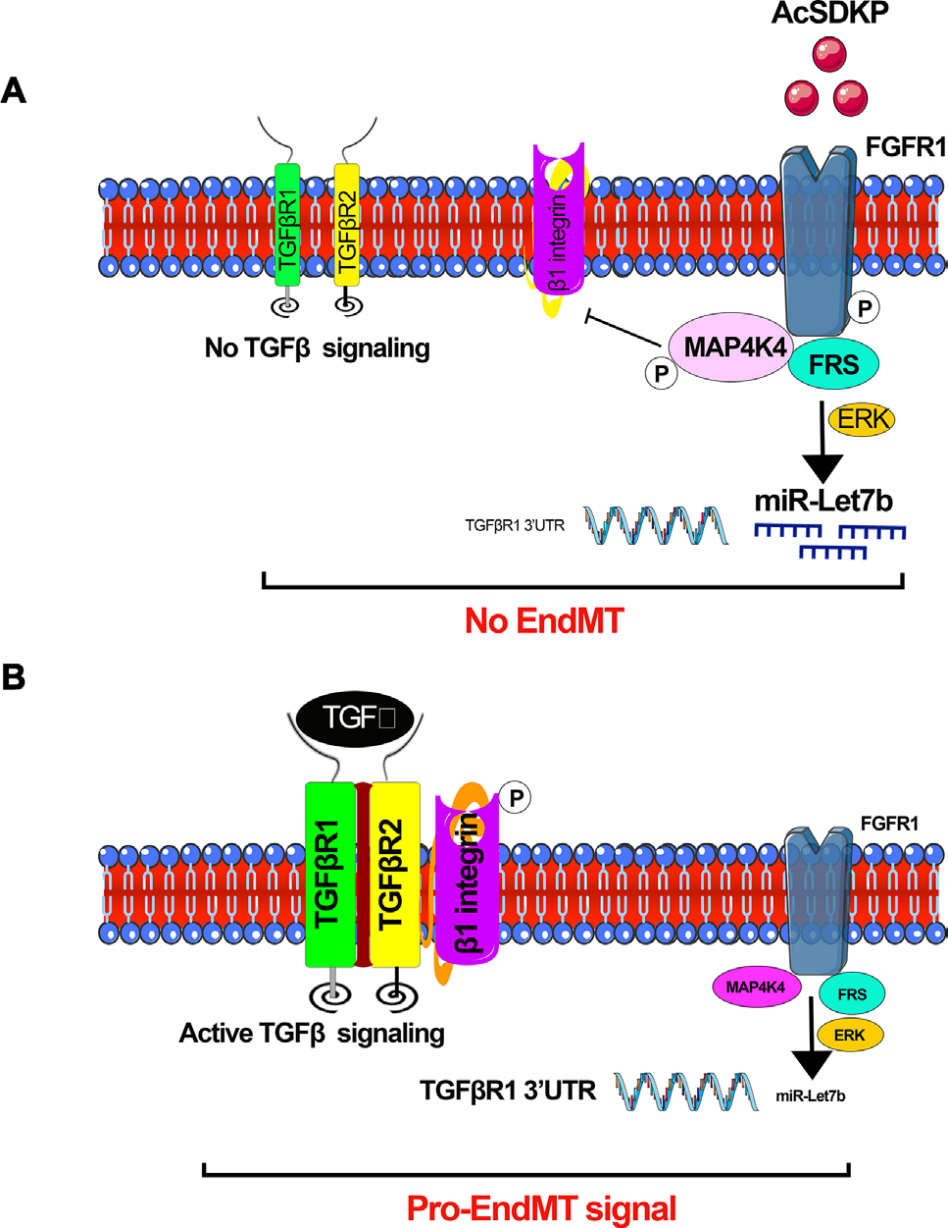
AcSDKP-mediated regulation of active TGFβ signaling and EndMT processes. **(A)** Phosphorylation of FGFR1 is the key mechanism for AcSDKP action. Phosphorylation of FGFR1 leads to activation of MAP4K4 signaling and miR-let-7 production. miR-let-7b targets the 3’UTR of TGFβR1 mRNA. AcSDKP-associated production of miR-let-7 family clusters and activation of MAP4K4 negatively regulates the DPP-4 integrin β1-associated active TGFβ signaling in the endothelial cells. **(B)** In the absence of AcSDKP, reduced FGFR1 phosphorylation leads to down-regulation of miR-let-7b gene expression and suppressed MAP4K4 signaling, finally resulting in active TGFβ signaling and activation of pro-EndMT signals.

miR-let-7 family clusters demonstrate an anti-fibrotic role in lung fibrosis (Pandit et al., 2010; Rajasekaran et al., 2015), cardiac fibrosis (Wang et al., 2015), and renal fibrosis (Brennan et al., 2013; Srivastava et al., 2014; Srivastava et al., 2016). It was shown that TGFβ1 reinforces its signaling by mitigating miR-let-7b production, which targets the 3’UTR of TGFβR1 mRNA in rat TECs (Wang et al., 2014). Down-regulated miR-let-7b expression was found in mouse models of diabetic (Nagai et al., 2014) and non-diabetic renal fibrosis (Brennan et al., 2013). Similarly, miR-let-7c targets TGFβR1, collagen type 1 alpha 1 (COL1A1), collagen type 1 alpha 2 (COL1A2), and thrombospondin in human TECs (Brennan et al., 2013). Lipoxins, which are endogenously produced lipid mediators, decrease renal fibrosis in a UUO model in the rats by elevating miR-let-7c expression (Brennan et al., 2013), promote the resolution of inflammation, and inhibit fibrosis in cultured human proximal tubular epithelial (HK-2) cells (Brennan et al., 2013). Lipoxin A4 (LXA_4_) has been shown to decrease TGFβ1-induced expression of mesenchymal markers, i.e., fibronectin, N-cadherin, thrombospondin, and the notch ligand *jagged-1* in HK-2 cells through a mechanism by inducing of miR-let-7c (Brennan et al., 2013). In the UUO model of renal fibrosis, the expression level of miR-let-7c was up-regulated by treatment with LXA_4_ analog. LXA_4_ treatment caused up-regulation of miR-let-7c and inhibited TGFβR1 and its associated signaling. Therefore, LXA_4_-associated up-regulation of miR-let-7c expression suppresses TGFβ1-induced fibrosis, which is a key pathway that is dysregulated in human renal fibrosis. We discussed the role of lipid mediators in diabetic nephropathy in our previous published review (Srivastava et al., 2014). Protein kinase C (PKC) activation and ceramides are associated with the suppression of antifibrotic microRNAs, and cumulative effects lead to the induction of fibrogenic processes in the kidney; several anti-dyslipidemic drugs have a differential effect on renal outcome (Srivastava et al., 2014). AcSDKP inhibits EndMT-driven renal fibrosis by ameliorating the miR-let-7 family clusters (Nagai et al., 2014; Nitta et al., 2016; Li et al., 2017) and the miR-let-7s-FGFR1 axis inhibits TGFβ signaling in fibrotic kidneys (Nagai et al., 2014). AcSDKP inhibits TGFβ–smad3 signaling and EndMT *via* activation of the fibroblast growth factor receptor 1 (FGFR1)–mitogen-activated protein kinase kinase kinase kinase 4 (MAP4K4) pathway (Li et al., 2017). AcSDKP-associated induction of MAP4K4 signaling inhibits integrin β1 phosphorylation, leading to anti-EndMT signals (Li et al., 2017). AcSDKP exerts anti-EndMT and antifibrotic effects in several mouse models of organ fibrosis (Nagai et al., 2014; Nitta et al., 2016; Srivastava et al., 2016). However, the precise molecular mechanisms by which AcSDKP suppresses TGFβ–smad3 signaling and EndMT are not fully investigated. FGFR1 is a key inhibitor of TGFβ-induced EndMT (Chen et al., 2014b). FGFR1 is critical in the AcSDKP-induced suppression of TGFβ-associated EndMT by elevating the level of miR-let-7 family clusters (Nagai et al., 2014). AcSDKP-associated activation of MAP4K4 suppresses DPP-4–integrin β1 signaling in endothelial cells (Vitorino et al., 2015) and DPP-4–integrin β1 influences TGFβ signaling and EndMT (Shi et al., 2015). MAP4K4 is a crucial downstream protein responsible for the anti-EndMT effect of AcSDKP (Li et al., 2017). **Figure 4** depicts the contribution of interactions among AcSDKP, FGFR1, miR-let-7 family clusters, and MAP4K4 in endothelial cell homeostasis. The interaction between AcSDKP and FGFR1 mitigates the TGFβ–smad3 signaling associated EndMT by activating the MAP4K4 signaling pathway and by inducing miR-let-7 production. AcSDKP restores both diabetes-suppressed FGFR1 and MAP4K4 phosphorylation levels. The AcSDKP–FGFR1–MAP4K4 signaling axis offers significant information towards the understanding of endothelial cell homeostasis and provides a future target for the study of EndMT-associated organ fibrosis.

## Antifibrotic Crosstalk Regulation Between miR-29 and miR-let-7 Family Clusters

**Figure 5:**
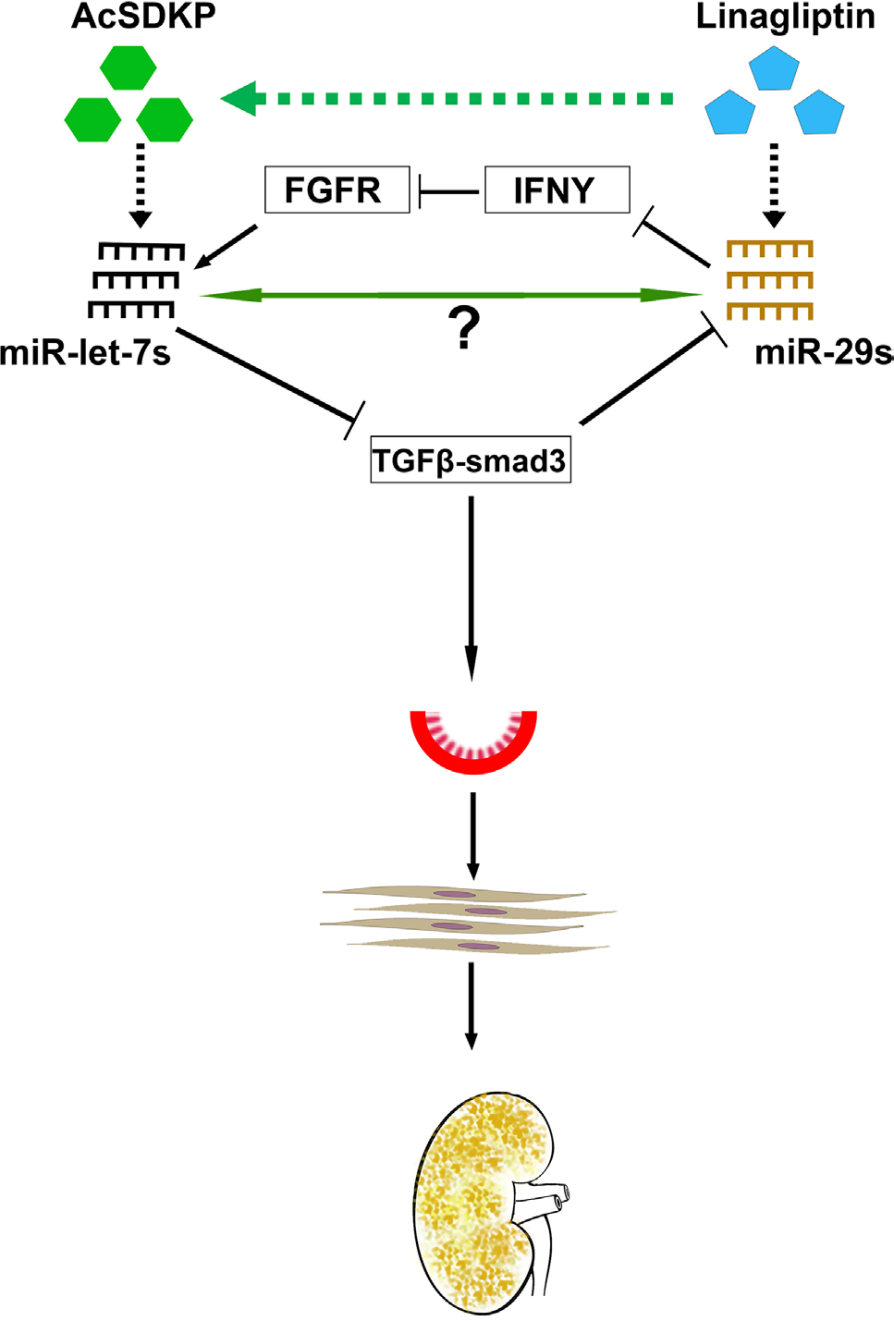
Antifibrotic microRNA crosstalk between miR-29s and miR-let7s is crucial for endothelial cell homeostasis. MiR-29 and miR-let-7 families show crosstalk regulation by inducing FGFR1 phosphorylation and targeting TGFβR1. AcSDKP potentiates crosstalk regulation in the endothelial cells, which is required for endothelial cell homeostasis. DPP-4 inhibition and/or AcSDKP elevate the crosstalk regulation in the endothelial cells. AcSDKP induces the production of miR-let-7 families; miR-let-7b targets TGFβR1 and TGFβ signaling. Suppressed levels of TGFβ signaling results in up-regulation of miR-29 gene expression, which in turn causes the FGFR1 phosphorylation. FGFR1 phosphorylation is critical for miR-let-7 production. In the presence of higher DPP-4 activity level or absence of AcSDKP, miR-let-7 families are down-regulated, which in turn causes activation of TGFβ signaling. Higher levels of TGFβ signaling results in suppression of miR-29 family expression and finally influences EndMT and fibrogenesis.

Previous reports show that TGFβ down-regulates anti-fibrotic miRNAs such as miR-29 family clusters (Wang et al., 2012a). TGFβ1-regulated crosstalk of miRNAs was de-regulated early in type 1 diabetes subjects who had accelerated rates of progression to ESRD (Pezzolesi et al., 2015). In addition, clusters of the miR-29 family and the miR-let-7 family displayed crosstalk regulation. AcSDKP is a key peptide for the homeostasis of this crosstalk in HMVECs (Srivastava et al., 2016) (**Figure 5**). Interestingly, miR-29 family clusters have shown negative, bidirectional regulation with TGFβRs. miRNAs could be regulating gene expression of each other directly or indirectly. Such a novel crosstalk phenomenon could be associated with maintenance of an anti-fibrotic milieu in the kidney, and disruption of such a mechanism could accelerate renal fibrosis. Pharmacological interventions that prevent the disruption of this crosstalk may be beneficial in renal fibrosis. The DPP-4 inhibitor (linagliptin) has been shown to suppress EndMT-driven TGFβ signaling in STZ-induced renal fibrosis in diabetic CD-1 mice by inducing miR-29 family clusters (Kanasaki et al., 2014). miR-29 directly targets DPP-4; therefore, DPP-4 inhibition is proposed for the therapy of diabetic nephropathy (Kanasaki et al., 2014). The development of renal fibrosis in mice is largely dependent upon strain types (Srivastava et al., 2018). The CD-1 mouse is well-known as a fibrotic mouse strain, while 129sv and C57Bl6 mouse strains are less fibrotic (Srivastava et al., 2016; Srivastava et al., 2018). The suppression of miR-29 and miR-let-7 family clusters and the induction of TGFβ–smad3 signaling were observed in the fibrotic kidneys of diabetic CD-1 mice; however, such alterations were not observed in the less fibrotic kidneys of diabetic 129sv mice, suggesting that miR-29 and miR-let-7 family clusters play key roles in regulation of TGFβ signaling (Srivastava et al., 2016).

MiR-let-7 has been shown to inhibit TGFβR1 (Chen et al., 2012), and TGFβ–smad3 signaling has been demonstrated as an inhibitory pathway of miR-29 (Qin et al., 2011; Blahna and Hata, 2012; Wang et al., 2012a; Kanasaki et al., 2014); therefore, it was expected that miR-let-7 could induce the expression level of miR-29. An alternative mechanism of miR-29-associated miR-let-7 expression was explained by the interferon-gamma (IFNγ)–FGFR1 axis. miR-29 targets IFN-γ mRNA (Ma et al., 2011). However, IFN-γ has been shown to inhibit FGFR1. FGFR1 exhibits vital roles in the production of miR-let-7 family clusters (Chen et al., 2012). Suppressed miR-29 causes elevation of IFN -γ; subsequently, higher levels of synthesized IFN-γ discourage FGFR1 and FGFR1-associated expression of miR-let-7 family clusters. This suppression of miR-let-7 causes induction of TGFβR1 protein expression. Triggering TGF-β/smad3 signaling, in turn, inhibits the expression of miR-29 family clusters (Kanasaki et al., 2014). This series of events limits control over the crosstalk regulation between miR-29 and miR-let-7 during fibrotic events in kidneys of diabetic mice. AcSDKP contributes to kidney homeostasis, at least in part, by maintaining the anti-fibrotic crosstalk regulation between miR-29 and miR-let-7.

## miR-192 and miR-200

TGFβ1-linked renal fibrosis has been shown to associate with miR-192 and miR-200; however, TGFβ1 has shown inconsistent effects on miR-192 expression in various *in vivo* and *in vitro* models (Meng et al., 2015). TGFβ1 has been shown to have diverse regulation of miR-192 in cultured mesangial cells and cultured TECs (Kato et al., 2007; Chung et al., 2010; Wang et al., 2010; Putta et al., 2012). Likewise, higher expression levels of miR-192 were found in fibrotic kidneys of mice after UUO (Chung et al., 2010). Conversely, lower expression of miR-192 was found in the fibrotic kidneys from a rat 5/6 nephrectomy model (Chung et al., 2010; Sun et al., 2011); however, the expression level of miR-192 was both up-regulated and down-regulated in experimental mouse models of diabetic nephropathy (Kato et al., 2007; Wang et al., 2010; Putta et al., 2012). These conflicting results were due to variations in the animal models, differences in the disease stage analyzed, and/or the diverse *in vitro* experimental conditions used. In the early stages, biphasic induction of miR-192 by TGFβ1 in mouse mesangial cells involves the smad dependent pathway, which is followed by an induction of the concomitant mechanism that causes expression miRNA by loosening the compacted chromatin structure of the miR-192 gene *via* Ets1 and histone H3 acetylation (Kato et al., 2013). An identical finding was shown in the glomeruli of db/db mice. In contrast, normal levels of miR-192 expression are found in mouse mesangial cells and human TECSs. In this case, both hepatocyte nuclear factor (HNF) and p53 constitutive binding regions are present in its promoter. TGFβ1 suppresses miR-192 transcription by reducing the binding between HNF and the miR-192 gene (Jenkins et al., 2012). HNF expression is restricted to the tubular compartment; however, it is expressed neither in mesangial cells nor in podocytes (Igarashi et al., 2005), thus substantiating the cell-specific regulation of miR-192.

TGFβ1 treatment of mouse mesangial cells and glomeruli from diabetic mice leads to up-regulation of miR-192 and miR-200b/c expression; TGFβ1 treatment causes induction of *Col1a2* and *Col4a1* by suppressing the E-box repressors Zeb1 and Zeb2 (Kato et al., 2007; Kato et al., 2011; Putta et al., 2012). Clinical studies in Southwestern American Indians with type 2 diabetes have suggested that expression of miR-192 inversely correlates with Zeb1 and Zeb2 expression levels (Deshpande et al., 2013). In contrast, TGFβ1-induced down-regulation of the miR-200 family (Tang et al., 2013), miR-192 (Krupa et al., 2010; Wang et al., 2010), and miR-215 (Krupa et al., 2010) causes diminished levels of E-cadherin (as miRNA targets E-cadherin transcriptional repressors Zeb1 and Zeb2) in TECs, UUO models of fibrosis (Jenkins et al., 2012), and diabetic models of fibrosis (Wang et al., 2010).

miRNA-regulated circuits in mouse mesangial cells and in the glomeruli of diabetic mice cause amplification of TGFβ1 signaling by forming an auto-regulatory loop involving TGFβ1, miR-192, and miR-200 family members (Kato et al., 2011). TGFβ1 induces crosstalk between p53 and miR-192. Since miR-192 targets Zeb2, this crosstalk has been explained as an auto-regulatory loop in mesangial cells and glomeruli from the kidneys of diabetic mice (Deshpande et al., 2013).

## Clinical Development of miRNA-Based Therapeutics

To date, around 20 clinical trials have been launched using miRNA and siRNA-based therapeutics against several diseases (Chakraborty et al., 2017). SPC3649 (miravirsen, Santaris Pharama Denmark), which is an antagomir of miR-122, is the only miRNA-based therapeutic available for the treatment of hepatitis C virus infection (Janssen et al., 2013; Gebert et al., 2014). In recent years, therapeutic microRNAs are some significant biopharmaceuticals that are (or will be) in the commercial space as future medicine for the treatment of kidney diseases (Brandenburger et al., 2018). A recent advance in miRNA-based therapeutics (RG-012) is now in the pipeline to initiate a phase 2 clinical trial. RG-012 (anti-miR-21) is being developed by Regulas Therapeutics for the treatment of Alport nephropathy and its complication (Chau et al., 2012; Gomez et al., 2015).

Several issues have been noticed during the design of miRNA-based therapeutics related to the absorption, distribution, metabolism, and excretion (ADME) of new chemical molecules (Caldwell, 2000; Ruiz-Garcia et al., 2008). miRNA-based therapies are often less efficient candidates in terms of absorption (Khatsenko et al., 2000); a more efficient delivery system and more research is needed. Importantly, the delivery of miRNA-based therapy to the target tissues is challenging because of poor pharmacological properties including off targeting, low serum stability, and poor innate immune response (Miller, 2013; Hong and Nam, 2014). Recent advances in the available delivery systems of miRNA-based therapy, such as PEGylated liposome vesicles, are 50–100 nm, which prevents the medicine from being filtered by the kidneys (Love et al., 2010; Broderick and Zamore, 2011; Hong and Nam, 2014). Liposomal encapsulation technology can improve the half-life of therapeutic miRNAs in blood; this is an area of active research and development at pharmaceutical companies.

## Perspective and Future Directions

Some miRNAs display down-regulated status in kidney disease, suggesting protective roles. Anti-fibrotic mechanisms of miRNAs could be dependent on signaling molecules in TGFβ pathways or independent from TGFβ pathways, i.e., targeting signaling molecules of ECM-secreting pathways. miRNA-based therapeutics are superior to those of conventional drug approaches because they are able to target complex pathogenic gene networks. Further benefits include sustained outcomes, expansion of drug-ridden targets to virtually any miRNA, rapid drug development, and limited potential for drug interactions (Pottier et al., 2014; Morishita et al., 2015). Using efficient delivery methods such as liposome-based delivery or nanoparticle-based delivery systems can minimize both the dose required and the toxicity level, both of which could be beneficial for the treatment of kidney diseases.

microRNAs can be used as biomarkers and therapeutic targets for kidney diseases (Fujii et al., 2019; Nascimento and Domingueti, 2019). The challenges to translate their therapeutic potential to clinical applications are a subject of ongoing research. miRNA-based therapies offer a significant promise for the treatment of kidney diseases. miR-let-7c-5p and miR-29a-3p were significantly linked with protection against rapid progression of renal fibrosis, whereas miR-let-7b-5p and miR-21-5p were linked with higher risk of ESRD. Controlling HgbA1c and other covariates, miR-let-7c-5p and miR-29a-3p were associated with significant (>50%) decline in increased progression, whereas miR-let-7b-5p and miR-21-5p were linked with more than a 2.5-fold higher rapid risk of ESRD (Pezzolesi et al., 2015). Some microRNAs need further investigation to establish their potential. miR-200b pre-cursor has been shown to be anti-fibrotic and its mimic can ameliorate renal interstitial fibrosis in UUO kidneys (Oba et al., 2010). Similarly, urinary expression of levels of miR-29b and miR-29c is linked to proteinuria and kidney function in immunoglobulin A (IgA) nephropathy, while urinary levels of miR-93 are coordinated with glomerular scarring (Wang et al., 2012b). MicroRNAs regulating the M2-to-M1 phenotype macrophages regulate MMT processes in kidneys. MiR-9, miR-125b, miR-127, and miR-155 induce the M1 polarization, whereas miR-124, miR-233, miR-34a, miR-132, miR-146a, and miR-125a induce M2 polarization (Essandoh et al., 2016). MicroRNAs regulating the TGFβ signaling (miR-let-7 family and miR-29 family) or smad3 dependent suppression in the antifibrotic microRNAs can be crucial in the regulation of MMT processes. Moreover, this new area needs further investigation.

**Figure 6:**
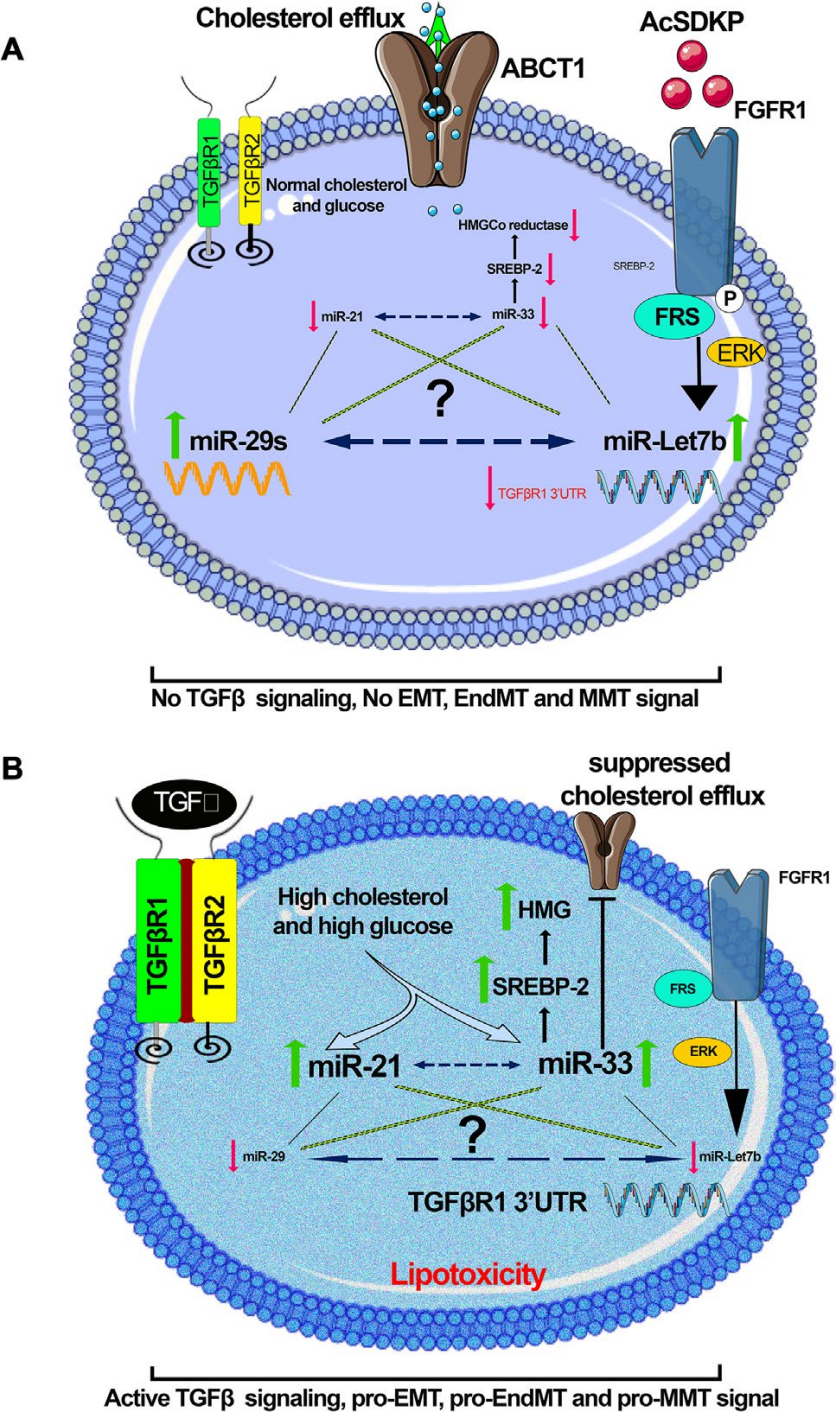
A hypothetical schematic diagram demonstration. **(A)** Anti-fibrotic; in the presence of AcSDKP, normal glucose, and lipid level, miR-29 and let-7 are found at normal expression level, whereas the expression level of miR-21 and miR-33 is down-regulated. **(B)** Profibrotic; in the absence of AcSDKP, the presence of hyperglycemia and hyperlipidemia suppresses the expression level of miR-29 and miR-let-7 but induces the expression level of profibrotic microRNA (miR-21 and miR-33) and influences mesenchymal activation in epithelial cells, endothelial cells, and M2 phenotype macrophages. Hyperglycemia is linked with up-regulation in the expression level of miR-21, whereas hyperlipidemia is found to be associated with miR-33. There would be a possibility that a kind of crosstalk mechanism exists among anti-fibrotic and pro-fibrotic microRNAs, which influences the EMT, EndMT, and MMT processes, and the endogenous peptide AcSDKP regulates such profibrotic mechanisms. Figures were created using the Servier Medical Art illustration resources.

Altered metabolic states can alter the expression level of pro-fibrotic and anti-fibrotic microRNAs. **Figure 6** depicts a hypothetical representation showing possible crosstalk among pro-fibrotic miRNAs (miR-33 and miR-21) and anti-fibrotic microRNAs though which mesenchymal activation is regulated. Hyperglycemia and hyperlipidemia up-regulate pro-fibrotic miRNAs, which could be a result of up-regulated TGFβ/smad3 signaling (Zhong et al., 2011; Chau et al., 2012; Kumarswamy et al., 2012; Nishiga et al., 2017). TGFβ signaling has been shown to up-regulate miR-21 (Liu et al., 2016; Kolling et al., 2017; Schauerte et al., 2017; Chau et al., 2012; Wang et al., 2012c; Kim, 2018; Sun et al., 2018) and down-regulate miR-29 (Qin et al., 2011). Identification of novel miRNA crosstalk mechanisms in the kidney is quite relevant to the understating of renal health and disease. Restoring anti-fibrotic miRNA crosstalk mechanisms provides renal protection. Physiologically relevant anti-fibrotic crosstalk may potentially be useful in combating diabetic kidney disease.

## Author Contributions

SS wrote the manuscript, made the figures, and provided intellectual output. AH helped in editing. KK and JG provided intellectual output in the manuscript.

## Funding

JG is supported by the National Heart, Lung and Blood Institute Grant R01-HL-131952.

## Conflict of Interest Statement

The authors declare that the research was conducted in the absence of any commercial or financial relationships that could be construed as a potential conflict of interest.
