# Erratum: microRNA Crosstalk Influences Epithelial-to-Mesenchymal, Endothelial-to-Mesenchymal, and Macrophage-to-Mesenchymal Transitions in the Kidney

**DOI:** 10.3389/fphar.2020.00011

**Published:** 2020-01-10

**Authors:** Frontiers Production Office

**Affiliations:** Frontiers Media SA, Lausanne, Switzerland

**Keywords:** microRNAs, diabetic kidney disease, kidney fibrosis, microRNA crosstalk, epithelial-to-mesenchymal transition, endothelial-to-mesenchymal transition, macrophage-to-mesenchymal transition

Due to a production error, the author name “Fahim Ahmad Hedayat” should be “Ahmad F. Hedayat”.

The publisher apologizes for this mistake. The original version of this article has been updated.

